# Long‐term outcome of intraventricular conduction delays in the general population

**DOI:** 10.1111/anec.12788

**Published:** 2020-08-17

**Authors:** Jani Rankinen, Petri Haataja, Leo‐Pekka Lyytikäinen, Heini Huhtala, Terho Lehtimäki, Mika Kähönen, Markku Eskola, Andrés Ricardo Pérez‐Riera, Antti Jula, Harri Rissanen, Kjell Nikus, Jussi Hernesniemi

**Affiliations:** ^1^ Faculty of Medicine and Health Technology Tampere University, and Finnish Cardiovascular Research Center Tampere Finland; ^2^ Heart Center Department of Cardiology Tampere University Hospital Tampere Finland; ^3^ Department of Clinical Chemistry Tampere University Hospital, and Fimlab Laboratories Tampere Finland; ^4^ Faculty of Social Sciences Tampere University Tampere Finland; ^5^ Department of Clinical Physiology Tampere University Hospital Tampere Finland; ^6^ Design of Studies and Scientific Writing Laboratory ABC School of Medicine São Paulo Brazil; ^7^ The Finnish Institute for Health and Welfare Helsinki Finland

**Keywords:** bundle branch block, electrocardiography, intraventricular conduction delay, population study, prognosis

## Abstract

**Background:**

Previous population studies have presented conflicting results regarding the prognostic impact of intraventricular conduction delays (IVCD).

**Methods:**

We studied long‐term prognostic impact and the association with comorbidities of eight IVCDs in a random sample of 6,299 Finnish subjects (2,857 men and 3,442 women, mean age 52.8, *SD* 14.9 years) aged 30 or over who participated in the health examination including 12‐lead ECG. For left bundle branch block (LBBB) and non‐specific IVCD (NSIVCD), two different definitions were used.

**Results:**

During 16.5 years’ follow‐up, 1,309 of the 6,299 subjects (20.8%) died and of these 655 (10.4%) were cardiovascular (CV) deaths. After controlling for known clinical risk factors, the hazard ratio for CV death, compared with individuals without IVCD, was 1.55 for the Minnesota definition of LBBB (95% confidence interval 1.04–2.31, *p* = .032) and 1.27 (95% confidence interval 0.80–2.02, *p* = .308) for the Strauss’ definition of LBBB. Subjects with NSIVCD were associated with twofold to threefold increase in CV mortality depending on the definition. While right bundle branch block, left anterior fascicular block and incomplete bundle branch blocks were associated with seemingly higher mortality, this was no longer the case after adjustment for age and sex. The presence of R‐R’ pattern was not associated with any adverse outcome.

**Conclusions:**

In a population study with long‐term follow‐up, NSIVCD and Minnesota definition of LBBB were independently associated with CV mortality. Other IVCDs had no significant impact on prognosis. The prognostic impact of LBBB and NSIVCD was affected by the definition of the conduction disorder.

## 1 INTRODUCTION

The clinical significance of various intraventricular conduction delays (IVCD) depends on the type of the conduction disorder and on the studied patient population. Both right (RBBB) and left bundle branch blocks (LBBB) are associated with adverse outcome in subjects with overt cardiovascular disease (CV; Wang et al., 2008; Zhang et al., 2012). In subjects with IVCDs without other evidence of cardiac disease (isolated bundle branch block), published reports show conflicting results. Some authors showed that RBBB was associated with increased all‐cause mortality, while other investigators found no effect on outcome (Bussink et al.., 2013; Haataja et al., 2015). The results of studies evaluating the prognostic impact of LBBB on all‐cause mortality in subjects without known CV disease are also somewhat conflicting (Haataja et al., 2015; Imanishi et al., 2006; Schneider, Thomas, Kreger, McNamara, & Kannel, 1979), and even the standard electrocardiographic (ECG) criteria for LBBB have been challenged (Strauss, Selvester, & Wagner, 2011). On the other hand, non‐specific IVCD (NSIVCD) is considered as an ECG marker of adverse outcome due to its potential association with structural heart disease (Eschalier et al., 2015; Haataja et al., 2015). The effect of the ECG definitions of LBBB and NSIVCD on outcome has not been reported in prior population studies.

Left anterior fascicular block (LAFB) is usually regarded as a conduction disorder without clinical significance if encountered in asymptomatic individuals (Elizari, Acunzo, and Ferreiro, 2007). Isolated left posterior fascicular block (LPFB) is a rare conduction disorder with no clear consensus on prognostic significance without CV disease (Pérez‐Riera et al., 2018). Previous scientific literature does not provide much information about the prevalence or prognostic significance of incomplete bundle branch blocks in individuals apparently free of CV disease. Somewhat surprisingly, one previous study found that incomplete RBBB (iRBBB) was associated with increased all‐cause and CV mortality (Haataja et al., 2015). Only two prior population studies have assessed the clinical significance of incomplete LBBB (iLBBB) and found no relation to CV mortality (Haataja et al., 2015); (Tervahauta, Pekkanen, Punsar, & Nissinen, 1996).

While the current guidelines suggest the use of transthoracic echocardiography to rule out structural heart disease in isolated LBBB, the recommendation is less stringent in patients with conduction disorders other than LBBB (Kusumoto et al., 2018). These recommendations are based on observational evidence, and due to the limited data, there is no consensus on the need of follow‐ups after the initial screening.

The purpose of this study was to explore the prevalence, relation to CV comorbidities and prognostic significance of IVCDs in a predominantly Caucasian general population during a total follow‐up time of 16.5 years.

## 2 METHODS

### 2.1 Study population

The Health 2000 is a major Finnish health examination survey. The survey was carried out in 2000–2001, and a representative stratified random cluster sample of the Finnish population was examined. For the population aged ≥ 80 years, the sampling probability was twice as high as among those <80 years. The implementation of the survey was described in detail elsewhere (Heistaro, 2000).

The Health 2000 sample comprised random sample of 8 028 individuals (3 637 men and 4 391 women) aged 30 or older, of whom 79% (6 354 individuals; 2 876 men and 3 478 women) participated in the health examination. After a home interview, a comprehensive health examination, including questionnaires, measurements (e.g., blood pressure and resting ECG), and physician's physical examination, was performed. The National Care Register for Health Care and the national register on rights to reimbursements for medication costs were linked to the Health 2000 Survey data. The study protocol of the Health 2000 survey was approved by the Epidemiology Ethics Committee of the Helsinki and Uusimaa Hospital District. The participants in the survey signed an informed consent both before the health interview and at the beginning of the health examination.

### 2.2 Definition of coronary heart disease and myocardial infarction

Classification as coronary heart disease (CHD) required at least one of the following: diagnosis of myocardial infarction (MI) and/or angina pectoris during the field health examination by a physician, large Q waves in the resting ECG, hospitalization for CHD (International Classification of Diseases [ICD]‐8 or ICD‐9 codes 410–414 or ICD‐10 codes I20–I25), a history of coronary revascularization procedure, the right to drug reimbursements for CHD, or the use of nitroglycerine combined with an anticoagulant, acetyl salicylic acid, or beta‐blocker. The Finnish Care Register for Health Care has been shown to be valid in identifying major CHD events (Pajunen et al., 2005).

Classification for MI required either a clinical diagnosis of old MI by the examining physician, large Q waves in the resting ECG, or a previous discharge diagnosis of MI (ICD‐8 or ICD‐9 code 410 or ICD‐10 codes I21–I22). Old MI was defined as a positive history of the condition in the medical records or old MI in the ECG, or typical self‐reported history of MI treated in hospital. Large Q waves indicating probable previous MI included Minnesota codes (MC) 1.1–1.3.

### 2.3 Heart failure, stroke, and peripheral artery disease

Heart failure (HF) classification required a clinical diagnosis by the examining physician and either a previous discharge diagnosis of HF (ICD‐8 code 4,270, ICD‐9 code 428, or ICD‐10 code I50) or the right to drug reimbursements for HF. The classification for stroke required one or more discharge diagnoses of stroke (ICD‐8 codes 430–431, 433–434, ICD‐9 codes 430–434, or ICD‐10 codes I60, I61, I63). Classification for peripheral arterial disease (PAD) required a clinical diagnosis by the examining physician or previous hospitalization for PAD.

### 2.4 Other measurements, definitions, and laboratory tests

The health examination included measurements of height, weight, body mass index (BMI), and waist circumference. Blood pressure (BP) was measured with a mercury sphygmomanometer (Mercuro 300, Speidel & Keller) from the right arm. Hypertension was defined as a clinic BP ≥ 140/90 mmHg or right to drug reimbursements for hypertension. Diabetes mellitus was defined as a serum glucose level of 7.0 mM or greater or a history of the use of oral hypoglycemic agents or insulin therapy. Smoking was defined as frequent use of tobacco products. Laboratory tests included measurements for high‐density lipoprotein cholesterol, total cholesterol, triglyceride, and serum glucose. Low‐density lipoprotein cholesterol was calculated with the Friedewald formula.

### 2.5 ECG registration and analysis

Standard 12‐lead ECGs were recorded in the resting supine position by MAC 5000 recorders (Marquette Hellige) and stored as digital data on a Marquette MUSE CV 5B system (Marquette Hellige). All ECGs were read, and the computerized diagnoses and measurements corrected if needed, by a physician experienced with ECG before being stored in the database. ECG was recorded and printed using a paper speed of 50 mm/s. The maximal filter setting of the system (150 hertz) was used. The Minnesota coding was performed at the Institute of Cardiology, Kaunas Medical Academy, Lithuania, by two investigators who were blinded to the clinical data of the subject. ECGs were obtained successfully in 6 318 individuals (99%) who attended the health examination. Abnormalities identified visually in the ECG strips were coded in accordance with the Minnesota coding scheme (Pekkanen, Nissinen, Puska, Punsar, & Karvonen, 1989). The electrical recordings were analyzed by means of Magellan software program (Marquette Electronics Inc.). Nineteen ECGs were rejected owing to data lost in further processes, leaving 6 299 ECGs for analysis.

### 2.6 Follow‐up

Mortality information until the end of December 2015 (total follow‐up time 16.5 years, median 15.9 years) was gathered by linking the personal identity code from the Health 2,000 Survey database to the Care Register for Health Care and the Causes of Death register, maintained by Statistic Finland, which records 100% of deaths of Finnish citizens in Finland and nearly 100% abroad. Mortality information was available for all subjects.

### 2.7 Exclusion criteria

There was no exclusion of subjects based on ECG findings. Final analysis was performed with 6 299 subjects: 3 442 women and 2 857 men.

### 2.8 Definition of IVCDs

For the identification of different intraventricular conduction delays, both Minnesota codes and measurements based on the Magellan software program were used. Six of the conduction delays were classified according to the respective Minnesota classes: LBBB_MC_ (code 7–1), RBBB (code 7–2), iRBBB (code 7–3), non‐specific IVCD_MC_ (code 7–4), the R‐R’ pattern in either of leads V1, V2 with R’ amplitude ≤R (R‐R’) (code 7–5), and iLBBB (code 7–6). Two different definitions for LBBB and NSIVCD were used. The Strauss’ definition of LBBB was used (LBBB_STRAUSS_) to identify subjects with “strict” LBBB (Strauss et al., 2011). The Strauss definition of LBBB includes a QRS duration ≥140 ms for men and ≥130 ms for women, along with mid‐QRS notching or slurring in ≥2 contiguous leads. ECGs not meeting the criteria for LBBB_STRAUSS_ were defined as non‐specific IVCD_STRAUSS_. For LAFB, we used the following definition: frontal QRS axis between –30° and –90°, rS configuration in II, III, and aVF, and qR configuration in aVL, with a QRS duration <120 ms. LPFB was defined as frontal QRS axis > 120°, lead I rS configuration, leads II, III, and aVF qR configuration, and no pathological Q waves in leads II, III, aVF. The accuracy of the classification was checked by manual ECG analysis by three of the investigators (JR, PH, and KN). The classifications proved to be accurate.

### 2.9 Statistical analyses

The prevalence of IVCDs was established in six age groups: 30–44, 45–54, 55–64, 65–74, 75–84, and 85 or older. Proportions were compared with the chi‐square test or Fisher's exact test. The complex sampling design was taken into account by correcting for the oversampling of subjects over 80 years of age. Data were categorized into ten groups according to the presence and type of IVCD (eight IVCDs with two definitions for LBBB and NSIVCD). CV death was defined as primary and all‐cause death as secondary study endpoint. Survival to each endpoint was assessed using the Kaplan–Meier method. Age and sex adjustments were included. Hazard ratios (HR) were calculated by univariate and multivariate Cox regression model analysis. Multivariate analysis included the following parameters: age, sex, CHD, MI, HF, New York Heart Association class, hypertension, diabetes mellitus, smoking, BMI, and low‐density lipoprotein cholesterol. Death from non‐CV causes was considered as a competing event to CV death. To take into account this competing risk, a model according to the method of Fine and Gray subhazards model was applied. Statistical significance was based on *p* < .05.

## 3 RESULTS

**Figure 1:**
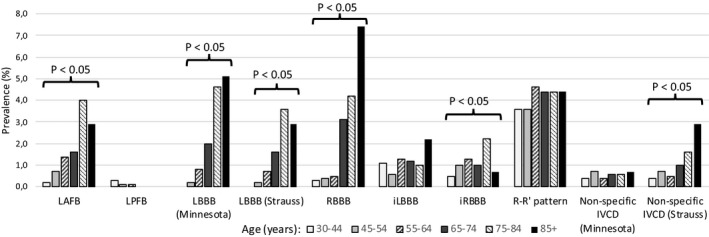
Prevalence of intraventricular conduction delays in six age groups; the significances of the difference within the age groups are shown (chi‐square test). iLBBB, incomplete LBBB; iRBBB, incomplete RBBB; IVCD, intraventricular conduction delay; LAFB, left anterior fascicular block; LBBB, left bundle branch block; LPFB, left posterior fascicular block; RBBB, right bundle branch block

Figure 1 (based on Supplemental Material) illustrates the prevalence of IVCDs divided by the six age groups. The prevalence of LAFB, LBBB, non‐specific IVCD_STRAUSS,_ and RBBB clearly increased with age, while for the other conduction delays, there was no clear age association. LBBB_STRAUSS_ criteria were met in 80% of subjects positive for LBBB_MC_.

Table 1 and Supplemental Material show the baseline and clinical characteristics. R‐R’, iRBBB, and LPFB had no clear relationship with CV diseases, while in subjects with LBBB and RBBB, there was a high prevalence of CV diseases and diabetes. The other IVCDs showed varied associations with risk factors and studied disease. LAFB, LBBB, NSIVCD, and RBBB were most strongly associated with HF, while LBBB, RBBB, NSIVCD, LAFB, and iLBBB were associated with the different manifestations of atherosclerosis.

**Table 1:** Clinical characteristics and mortality of the study population according to presence of intraventricular conduction delay

	Intraventricular conduction delay										
	No IVCD (*n* = 5 587)	LAFB (*n* = 69)	LPFB (*n* = 8)	LBBB_MC_ (*n* = 59)	LBBB_STRAUSS_ (*n* = 47)	RBBB (*n* = 75)	iLBBB (*n* = 66)	iRBBB (*n* = 61)	R‐R' pattern (*n* = 249)	Non‐specific IVCD_MC_ (*n* = 33)	Non‐specific IVCD_STRAUSS_ (*n* = 45)
	*n* (%)	*n* (%)	*n* (%)	*n* (%)	*n* (%)	*n* (%)	*n* (%)	*n* (%)	*n* (%)	*n* (%)	*n* (%)
Smoking (current)	1,549 (27.2)	10 (14.7)*	1 (12.5)	9 (15.3)*	8 (17.0)	15 (20.0)	18 (29.5)	12 (19.7)	71 (28.5)	6 (18.2)	7 (15.6)
Hypertension	2,671 (46.9)	50 (73.5)*	1 (12.5)	47 (79.7)*	37 (78.7)*	50 (66.7)*	35 (57.4)	31 (50.8)	115 (46.2)	22 (66.7)*	32 (71.1)*
Diabetes mellitus	324 (5.7)	6 (8.8)	0	10 (16.9)*	8 (17.0)*	9 (12.0)*	4 (6.6)	6 (9.8)	7 (2.8)*	2 (6.1)	4 (8.9)
Heart failure	118 (2.1)	9 (13.2)*	0	12 (20.3)*	9 (19.1)*	13 (17.3)*	3 (4.9)	1 (1.6)*	5 (2.0)	3 (9.1)*	6 (13.3)*
NYHA class II‐IV	355 (6.2)	10 (14.7)*	0	27 (46.6)*	19 (41.3)*	16 (21.6)*	6 (9.8)	8 (13.3)	10 (4.0)	5 (15.2)	13 (28.9)*
Stroke	213 (3.7)	6 (8.8)*	0	8 (13.6)*	4 (8.5)	6 (8.0)	1 (1.6)	1 (1.6)	14 (5.6)	5 (15.2)*	9 (20.0)*
Peripheral artery disease	82 (1.4)	2 (2.9)	0	6 (10.2)*	4 (8.5)*	3 (4.0)	1 (1.6)	5 (8.2)	4 (1.6)	3 (9.1)*	5 (11.1)*
Coronary heart disease	529 (9.3)	14 (20.6)*	0	31 (52.5)*	24 (51.1)*	25 (33.3)*	8 (13.1)	10 (16.4)	20 (8.0)	10 (30.3)*	17 (37.8)*
Myocardial infarction	189 (3.3)	4 (5.9)*	0	17 (28.8)*	11 (23.4)*	6 (8.0)*	5 (8.2)*	3 (4.9)	10 (4.0)	9 (27.3)*	15 (33.3)*
Death											
All‐cause	1,097 (19.2)	31 (45.6)*	1 (12.5)	37 (62.7)*	27 (57.4)*	45 (60.0)*	14 (23.0)*	21 (34.4)*	53 (21.3)	10 (30.3)	20 (44.4)*
Cardiovascular	435 (7.6)	17 (24.6)*	1 (12.5)	27 (45.8)*	20 (42.6)*	33 (44.0)*	10 (15.2)	9 (14.8)	31 (12.4)	9 (27.3)*	16 (35.6)*
Medication											
ACI/ARB	454 (8.0)	8 (11.8)	0	15 (25.4)*	12 (25.5)*	5 (6.7)	5 (8.2)	9 (14.8)*	17 (6.8)	8 (24.2)*	11 (24.4)*
Beta adrenergic blockers	794 (13.9)	15 (22.1)	1 (12.5)	25 (42.4)*	20 (42.6)*	20 (26.7)*	7 (11.5)	15 (24.6)*	40 (16.1)	12 (36.4)*	17 (37.8)*
Calcium channel blockers	313 (5.5)	4 (5.9)	0	9 (15.3)*	7 (14.9)*	10 (13.3)*	1 (1.6)	4 (6.6)	18 (7.2)	7 (21.2)*	9 (20.0)*
Antithrombotics	513 (9.0)	17 (25.0)*	0	19 (32.2)*	12 (25.5)*	20 (26.7)*	10 (16.4)	14 (23.0)*	26 (10.4)	11 (33.3)*	18 (40.0)*
Diuretics	391 (6.9)	9 (13.2)	0	16 (27.1)*	9 (19.1)*	19 (25.3)*	7 (11.5)	6 (9.8)	17 (6.8)	7 (21.2)*	14 (31.1)*
Statin	348 (6.1)	3 (4.4)	0	7 (11.9)	5 (10.6)	6 (8.0)	4 (6.6)	5 (8.2)	12 (4.8)	4 (12.1)	6 (13.3)*

Abbreviations: ACI, angiotensin‐converting enzyme inhibitor; ARB, angiotensin receptor antagonist; iLBBB, incomplete LBBB; iRBBB, incomplete RBBB; IVCD, intraventricular conduction delay; LAFB, left anterior fascicular block; LBBB, left bundle branch block; LPFB, left posterior fascicular block; MC, Minnesota definition; NYHA, New York Heart Association; RBBB, right bundle branch block; Strauss, Strauss definition.

* *p* < .05

### 3.1 Outcome

During 16.5 years’ follow‐up, 1,309 of the 6,299 subjects (20.8%) died and of these 655 (10.4%) were CV deaths. Table 2 shows the unadjusted mortality rates for the different IVCDs. For all‐cause mortality, subjects with LBBB, RBBB, LAFB, NSIVCD, iLBBB, and iRBBB had the highest mortality rates, while for CV deaths, the highest rates were found in the LBBB, RBBB, NSIVCD, and LAFB categories.

**Table 2:** Adjusted Cox proportional hazard analysis for cardiovascular mortality according to intraventricular conduction delay

Intraventricular conduction delay	Cardiovascular mortality								
	Unadjusted			Age‐ and sex‐adjusted			Multivariate*‐adjusted		
	Hazard ratio	95% CI	*p* Value	Hazard ratio	95% CI	*p* Value	Hazard ratio	95% CI	*p* Value
LAFB	2.76	1.68–4.53	<.001	0.94	0.66–1.34	.729	0.75	0.43–1.31	.318
LPFB	1.21	0.17–8.57	.852	6.96	0.98–49.73	.053	1.24	0.78–40.19	.088
LBBB_MC_	7.51	5.10–11.04	<.001	2.05	1.39–3.02	<.001	1.55	1.04–2.31	.032
LBBB_STRAUSS_	6.35	4.07–9.92	<.001	1.77	1.13–2.77	.012	1.27	0.80–2.02	.308
RBBB	6.28	4.42–8.93	<.001	1.31	0.92–1.87	.142	1.43	0.98–2.08	.066
iLBBB	1.02	0.54–1.90	.960	0.97	0.52–1.81	.922	0.56	0.29–1.10	.092
iRBBB	1.75	0.91–3.39	.095	1.16	0.60–2.24	.657	1.35	0.69–2.62	.379
R‐R' pattern	1.05	0.73–1.51	.779	0.94	0.66–1.36	.750	1.05	0.72–1.52	.806
Non‐specific IVCD_MC_	3.23	1.67–6.24	<.001	2.76	1.43–5.35	.003	2.30	1.85–4.49	.015
Non‐specific IVCD_STRAUSS_	4.96	3.02–8.15	<.001	3.15	1.91–5.18	<.001	2.87	1.72–4.78	<.001

Abbreviations: CI, confidence interval; iLBBB, incomplete LBBB; iRBBB, incomplete RBBB; IVCD, intraventricular conduction delay; LAFB, left anterior fascicular block; LBBB, left bundle branch block; LPFB, left posterior fascicular block; MC, Minnesota definition; RBBB, right bundle branch block; Strauss, Strauss definition.

* Adjusted for age, sex, coronary heart disease, myocardial infarction, heart failure, NYHA class, hypertension, diabetes mellitus, smoking, body mass index, and low‐density lipoprotein cholesterol.

In the age‐ and sex‐adjusted Cox regression analysis (Table 2), the HR for CV death for LBBB_MC_ was 2.05 (95% confidence interval 1.39–3.02, *p* < .001), for LBBB_STRAUSS_ 1.77 (1.13–2.77, *p* = .012), for non‐specific IVCD_MC_ 2.76 (1.43–5.35, *p* = .003) and for non‐specific IVCD_STRAUSS_ 3.15 (1.91–5.18, *p* < .001). In the multivariate‐adjusted Cox model, LBBB_MC_ and NSIVCD regardless of the definition retained their statistical significance to predict CV death.

LBBB_MC_, but not LBBB_STRAUSS_, was associated with all‐cause mortality in age‐ and sex‐adjusted Cox regression analysis (1.49, 1.07–2.07, *p* = .018), but not after multivariate adjustment. Subjects with non‐specific IVCD_STRAUSS_ were associated with all‐cause mortality both in age‐ and sex‐adjusted (2.07, 1.33–3.23, *p* = .001), and multivariate‐adjusted (2.01, 1.27–3.18, *p* = .003) Cox regression analysis. Subjects with non‐specific IVCD_MC_ displayed no relation to increased all‐cause mortality.

In the Cox regression analysis of subjects with history of heart disease (CHD, previous MI, or HF), after controlling for known clinical risk factors, subjects with NSIVCD, LBBB_MC,_ and iRBBB were associated with all‐cause and CV mortality, and subjects with RBBB were associated with CV mortality (see Supplemental Material).

## 4 DISCUSSION

The main findings of the present study were that NSIVCD and LBBB_MC_, but not LBBB_STRAUSS_, were associated with increased CV mortality after adjustment for baseline cardiac comorbidities. Regarding mortality, LBBB_STRAUSS_ identifies subjects with seemingly lower risk for death when compared to the LBBB_MC_ definition. However, subjects with NSIVCD had significantly worse outcome when compared to subjects with LBBB by the Strauss’ criteria. LAFB and iLBBB displayed relationship with mortality in unadjusted Cox regression analysis but neither impaired the prognosis after adjustments for age and sex.

The Framingham Heart Study (*n* = 5,209) described a close relation to CV diseases in LBBB patients (Schneider et al., 1979). In the present study, there was a high prevalence of CV diseases in subjects with LBBB, and 52.5% of the subjects had known CHD. In our subgroup analyses, LBBB was associated with higher CV mortality in subjects with history of heart disease. The Reykjavik Health Survey (*n* = 17,489; Hardarson et al., 1987) and the follow‐up study of atomic bomb survivors in Hiroshima and Nagasaki (*n* = 17,361; Imanishi et al., 2006) reported no increased all‐cause mortality in subjects with LBBB. In the Framingham Heart study, multivariate risk analysis indicated that the risk for incident CHD morbidity remained significant in women but not in men   (Schneider et al., 1979). In the Women's Health Initiative study (*n* = 68,133; Zhang et al., 2012), LBBB was associated with increased CV mortality in patients without known CV disease. Similarly, in the Primary Prevention Study from Gothenburg (*n* = 7,392), LBBB was a marker of adverse prognosis in symptom‐free men (Eriksson, Wilhelmsen, & Rosengren, 2005). Thus, LBBB may be a marker of a slowly progressing disease that not only affects the conduction system but also the myocardium itself (Eriksson et al., 2005). The differences in study results may be due to differences in the diagnostic level of baseline cardiac diseases and also to the patient populations studied.

LBBB_STRAUSS_ criteria were met in 80% of subjects positive for LBBB_MC_. The result is close to a previous population study (Almer et al., 2015), where the Strauss’ definition was met in 87% of LBBB patients. To our knowledge, this is the first study to investigate the influence of the definition of LBBB and NSIVCD on outcome in a nationally representative population. In the present study, LBBB_STRAUSS_ was associated with lower risk of death compared to LBBB_MC_. The finding is probably explained by the superiority of the LBBB_STRAUSS_ definition to sort out patients with NSIVCD from those with genuine conduction delay induced by the conduction disorder. This finding is in line with a previous cardiac resynchronization therapy study, which investigated the influence of the definition of LBBB in patients with HF. The study results showed that the Strauss’ definition was significantly better than other definitions of LBBB in predicting survival (Jastrzebski et al., 2018).

We found a strong independent association between NSIVCD and CV mortality even after adjustment for baseline cardiac comorbidities, and the association was strongest for non‐specific IVCD_STRAUSS_. Although less studied than LBBB and RBBB and probably under‐diagnosed by clinicians, there are studies showing a strong correlation between NSIVCD and CV mortality. Regional myocardial scarring as a result of fibrosis, left ventricular hypertrophy, or previous MI has been considered as pathophysiological background factors for NSIVCD (Eschalier et al., 2015; Haataja et al., 2015). This conduction disorder alters left ventricular conduction, which results in a broad QRS complex not typical for RBBB or LBBB. In the retrospective Palo Alto Veterans Affairs Medical Center study (*n* = 46,933), every 10 ms increase in QRS duration without bundle branch block increased CV risk by 18% (Desai et al., 2006). In a Finnish community‐based CHD Study (*n* = 10,899) carried out between 1966 and 1972, NSIVCD was a predictor of all‐cause and CV mortality with an increased risk of sudden arrhythmic cardiac death (Aro et al., 2011). In Women's Health Initiative study, NSIVCD was independently associated with increased CV mortality in women with known CV disease. In women without CV disease, NSIVCD was not a predictor of all‐cause mortality and CV mortality was not reported (Zhang et al., 2012). The results from the current study emphasize NSIVCD as a marker of increased mortality especially in subjects with prevalent heart disease.

Although RBBB had a frequent association with CV comorbidities in the present study, no relation to adverse prognosis was found in the general population. However, in subjects with prevalent heart disease, RBBB was associated with higher CV but not with all‐cause mortality. In the Copenhagen City Heart Study (*n* = 18,441; Bussink et al.., 2013), RBBB was associated with increased risk of all‐cause and CV mortality in subjects free from previous MI or HF but the prevalence of stable CHD was not reported. In the Women's Health Initiative study (Zhang et al., 2012), RBBB was associated with CV mortality only in women with CV disease at baseline, and likewise was not associated with mortality in subjects without angina or dyspnea at baseline in the Primary Prevention Study (Eriksson et al., 2005).

The data regarding prognosis of incomplete bundle branch blocks in general population are scarce. ILBBB is thought to result from slowing of conduction in the left bundle branch, and an association with CHD and hypertensive heart disease was found in a study from the 1960s (Wassenburger, White, & Lindsay, 1963). In the present study, iLBBB was associated with previous MI and was related to mortality only in unadjusted Cox regression analysis. Conversely, iRBBB was not associated with mortality in absence of heart disease, similar to the results of the Copenhagen Heart Study (Bussink et al.., 2013) and to an older Chicago Western Electric Company Study (*n* = 1,960; Liao et al., 1986). However, in exploratory subgroups analyses, we found that among subjects with heart disease iRBBB associated with increased and all‐cause mortality suggesting that iRBBB might not be a harmless finding. We found no prior prospective population studies regarding this matter. iRBBB has been associated with exercise‐induced physiological left ventricular remodeling and right ventricular enlargement (Kim et al., 2011), right ventricular pressure overload (Digby et al., 2015), and degenerative heart disease of the elderly (Bussink et al.., 2013). Thus, iRBBB observed in early life may be of a different etiology than in the elderly (Nielsen et al., 2011).

In epidemiological studies, the association of LAFB and CV diseases has shown varied results. In patients with suspected CHD and no history of MI (*n* = 1,187; Biagini et al., 2005), LAFB was associated with increased CV mortality. In the Kailuan study (*n* = 101,510; Yiheng et al., 2016), no association between LAFB and mortality was found. In the present study, no relation to adverse prognosis was found although LAFB was related to multiple cardiac comorbidities. Some overlap between left axis deviation and LAFB is unavoidable, and isolated left axis deviation is a common, age‐associated ECG finding not associated with adverse prognosis (Ostrander, 1971).

As in previous studies, LPFB was an infrequent IVCD in the present study. Anatomically, the left posterior fascicle is shorter and thicker than the left anterior fascicle. In addition, the posterior fascicle has double arterial blood supply (Elizari et al., 2007). LPFB is often encountered with RBBB (Godat & Gertsch, 1993) as a precursor of complete heart block (Boule et al., 2014; Elizari et al., 2007). Earlier studies associated LPFB with severe myocardial damage (Godat & Gertsch, 1993). However, the low number of subjects even in a large nationwide study makes it difficult to draw any firm conclusions about the clinical significance of this conduction disorder.

The R‐R’ pattern proved to be a benign ECG finding. In lead V1‐V2, the presence of R > R’ may be due to misplacement of the ECG electrodes in the 2nd intercostal place, especially when accompanied by a negative P wave in lead V1. In a previous study, the R‐R’ disappeared when the electrodes were properly positioned (Baranchuk et al., 2015). Another possible cause for this ECG manifestation is a normal variant due to delay in the activation of the basal part of the right ventricle (Baranchuk et al., 2015).

While the guidelines are less stringent in patients with conduction disorders other than LBBB, clinical evaluation and transthoracic echocardiography might be useful to rule out structural heart disease in subjects with NSIVCD. In isolated LBBB, the former is prudent (Kusumoto et al., 2018) as LBBB may not only indicate adverse prognosis but also have influence on the management of the heart disease. While bundle branch blocks may point to a greater degree of myocardial involvement and damage in subjects with prevalent heart disease, in some patients they may also indicate degeneration of the conduction system with no relation to impaired prognosis.

Several study limitations need to be pointed out. First of all, absence of imaging data is a study limitation typical of a population study. Furthermore, only one ECG for each subject was recorded. We also lack data related to possible changes in medication during follow‐up. We think that the large study population representing a wide age range from both genders, well‐defined baseline characteristics, and long follow‐up gives strength to our study findings.

In conclusion, in a population study of individuals aged 30 or older with long‐term follow‐up, LBBB and NSIVCD were associated with CV mortality. The definition of LBBB has influence on outcome. In further subgroup analyses, NSIVCD, LBBB, iRBBB, and RBBB were associated with mortality only in subjects with known heart disease. Other intraventricular conduction disorders had no significant impact on prognosis. These differences in the prognostic significance of different IVCDs need to be taken into account in everyday clinical practice.

## CONFLICTS OF INTEREST

None.

## ETHICAL APPROVAL

The study protocol of the Health 2000 survey was approved by the Epidemiology Ethics Committee of the Helsinki and Uusimaa Hospital District. The participants in the survey signed an informed consent both before the health interview and at the beginning of the health examination.

## Supporting information

Supplementary Material
